# Supplementary material for: Stratification in planetary cores by liquid immiscibility in Fe-S-H
Source: Nat Commun. 2022 Feb 3;13:644. doi: 10.1038/s41467-022-28274-z (PMC8813981; doi:10.1038/s41467-022-28274-z)
Supplement: Supplementary file 1 — Supplementary Information [file 41467_2022_28274_MOESM1_ESM.pdf]

Supplementary Information for

Stratification in planetary cores by liquid immiscibility in Fe-S-H

by Yokoo *et al*.

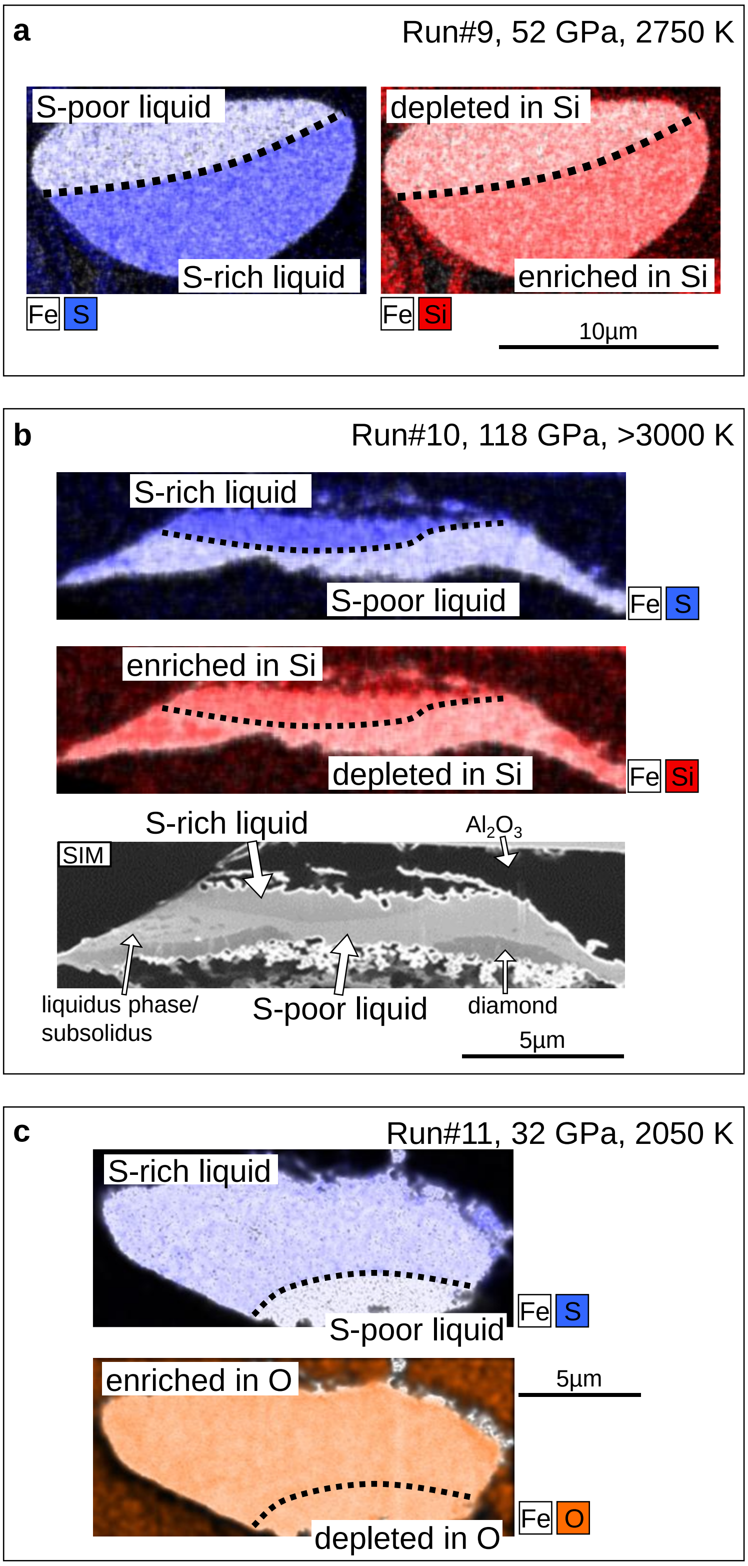

Supplementary Fig. 1 X-ray maps for Fe, S, Si and O concentrations in sample cross sections. (**a**) run #9, (**b**) run #10 and (**c**) run #11. Scanning ion microscope (SIM) image is also shown in **b**.

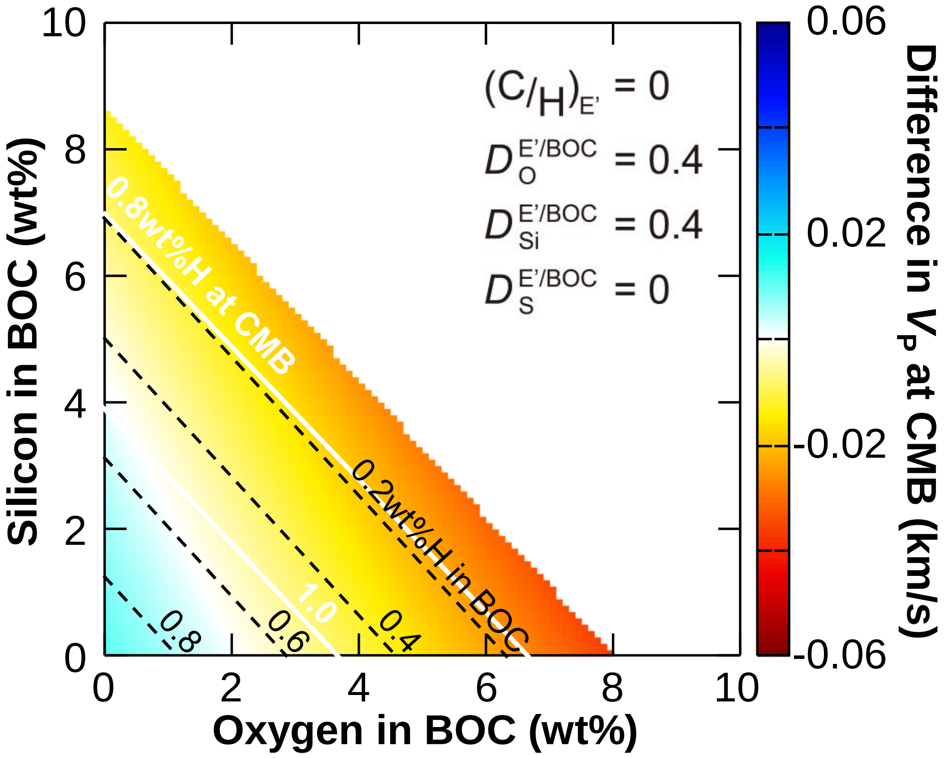

Supplementary Fig. 2 Difference in *P*-wave velocity (V_P) between S-rich bulk outer core (BOC) and H-rich E' layer with a smaller density. Conditions are the same as those for Fig. 4a except that the E' layer is lighter by 0.1 g/cm^3 than the BOC when compared at the core-mantle boundary.

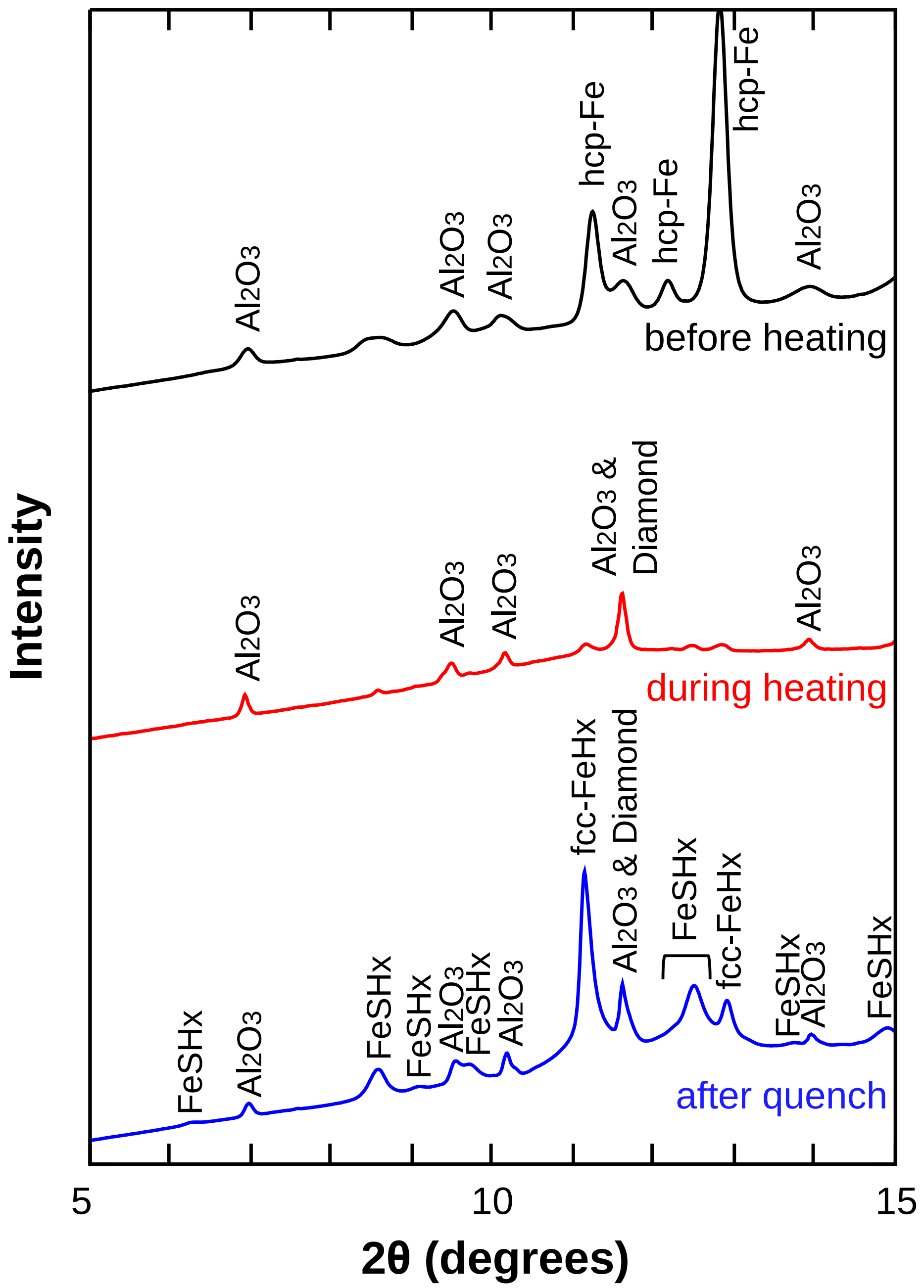

Supplementary Fig. 3 X-ray diffraction patterns indicating hydrogen abundance in Fe alloys. Data were collected before, during and after heating in run #2, showing the molten state of the sample during heating, and the appearance of FeH_x and $FeSH_x$ upon quenching temperature.

Supplementary Table 1 Experimental settings and results.

Miscible

Run#	Starting materials*	Quenched phases	x in FeH_x	x in $FeSH_x$	Averaged x
1	Fe10S+P, A	fcc-FeHx , FeSHx	1.18(13)	0.25(9)	1.04(11)
2	Fe10S+P, A	fcc-FeHx , FeSHx	1.05(10)	0.25(9)	0.79(7)
4	Fe10S+P, A	amorphous	-	-	-
5	Fe10S+P, A	dhcp- FeHx , FeSHx	1.08(11)†	0.20(15)	0.86(9)
6_1	Fe10S+P, A	fcc-FeHx , FeSHx	1.15(12)	0.15(10)	1.06(11)
12	Fe10S+P, A	fcc-FeHx , FeSHx	1.13(12)	0.24(12)	0.98(10)
13	Fe10S+P, A	not measured	-	-	-
14	Fe10S+P, A	not measured	-	-	-
15	Fe10S+P, A	not measured	-	-	-

Immiscible

Run#	Starting materials*	Quenched phases	x in FeH_x	x in $FeSH_x$	Averaged x
3	Fe10S+P	fcc-FeHx , FeSHx	1.06(11)	0.2(2)	-
6_2	Fe10S+P, A	-	-	-	-
7	Fe10S+P	amorphous	-	-	-
8	Fe10S+P, A	fcc-FeHx , Fe_7C_3	1.27(17)	-	0.40(8)
9	Fe2S3Si+P	hcp-FeHx	1.05(13)	-	-
10	Fe2S3Si+P, A	hcp-FeHx , Fe_7C_3	1.12(16)	-	0.56(11)
11	Fe7S5O+P	fcc-FeHx , FeSHx	1.14(13)	0.15(7)	-

Numbers in parentheses indicate errors (1σ) in the last digits.

*Fe10S = Fe-10.3wt%S, Fe2S3Si = Fe-2.1wt%S-2.5wt%Si, Fe7S5O = Fe-6.5wt%S-5.2wt%O, P = paraffin, A = Al_2O_3 pressure medium

†Volume of hcp Fe was used in Eq. (1).
